# Biomimetic behaviors in hydrogel artificial cells through embedded organelles

**DOI:** 10.1073/pnas.2307772120

**Published:** 2023-08-21

**Authors:** Matthew E. Allen, James W. Hindley, Nina O’Toole, Hannah S. Cooke, Claudia Contini, Robert V. Law, Oscar Ces, Yuval Elani

**Affiliations:** ^a^Department of Chemistry, Imperial College London, Molecular Sciences Research Hub, London W12 0BZ, UK; ^b^Department of Chemical Engineering, Imperial College London, South Kensington, London SW7 2AZ, UK; ^c^FabriCELL, Imperial College London, Molecular Sciences Research Hub, London W12 0BZ, UK

**Keywords:** artificial cells, hydrogels, artificial organelles, membranes

## Abstract

Artificial cells are biomimetic structures formed from molecular building blocks that replicate biological processes, behaviors, and architectures. Of these building blocks, hydrogels have emerged as ideal, yet underutilized candidates to provide a gel-like chassis in which to incorporate both biological and nonbiological componentry which enables the replication of cellular functionality. Here, we demonstrate a microfluidic strategy to assemble biocompatible cell-sized hydrogel-based artificial cells with a variety of different embedded functional subcompartments, which act as engineered synthetic organelles. The organelles enable the recreation of increasingly biomimetic behaviors, including stimulus-induced motility, content release through activation of membrane-associated proteins, and enzymatic communication with surrounding bioinspired compartments. In this way, we showcase a foundational strategy for the bottom–up construction of hydrogel-based artificial cell microsystems which replicate fundamental cellular behaviors, paving the way for the construction of next-generation biotechnological devices.

## Significance

Cellular form and functionality can be mimicked through entities known as artificial cells. One emerging chassis for artificial cells are hydrogel microparticles, which are gaining prominence due to their ability to replicate the gel-like environments present within cells, and more broadly, because of their biotechnologically useful properties. However, there are limited strategies to create sophisticated hydrogel-based artificial cells. Here we demonstrate a microfluidic production strategy for hydrogel artificial cells by incorporating a range of modular, interchangeable subcompartments into hydrogel microparticles. We then leverage this subcompartment toolkit to produce hydrogel artificial cells with a range of relevant biological behaviors including motility, sensing, and communication. This paves the way to the production of more sophisticated hydrogel artificial cells that can be used for a range of biotechnological applications.

Artificial cells have emerged as bioinspired platforms that can mimic the wide-ranging functionalities and behaviors of biological systems (1–3). Cellular features that have been mimicked are extensive, and include compartmentalization (4, 5), growth (6), communication (7), and motility (8). Furthermore, artificial cells have been integrated with living systems (9) and complex genetic circuitry (10) to expand the repertoire of replicable behaviors (11, 12). Artificial cells thus have tremendous potential for downstream industrial and clinical applications as smart carriers for drug delivery, synthetic cell therapies, diagnostic sensors, chemical microreactors, and as microscale biofoundries (13–16).

Biomimetic building blocks used to construct the chassis of artificial cells have included lipids (17), polymers (18), coacervates (19), and proteins (20, 21). Recently, hydrogels have emerged as an alternative chassis for constructing artificial cells due to their ability to more accurately mimic cellular environments that include the nucleus, cytoplasm, cytoskeleton, and extracellular matrix (22). Hydrogels can mimic these cellular attributes through possessing a 3-dimensional hydrophilic network, which holds a significant volume of bound water (23). This network provides reduced diffusion coefficients (24) and increased viscosities (25) compared to water, which are more representative of cellular environments (22) For instance, the cytoplasm has a viscosity ~1.2 larger and diffusion rates ~2 smaller than water (22). They are selectively permeable to small molecules but can encapsulate large proteins and enzymes (26), are biocompatible (27), can be engineered to respond to stimuli (28), and can controllably release stored substances (29)—all appealing features for constructing an artificial cell chassis. As a consequence, hydrogels have been extensively used in biotechnical fields which include tissue engineering (30), drug delivery (31), and biomaterials (32). These fields are not dissimilar to those that artificial cell technology is envisaged to have the greatest impact.

**Fig. 1.**
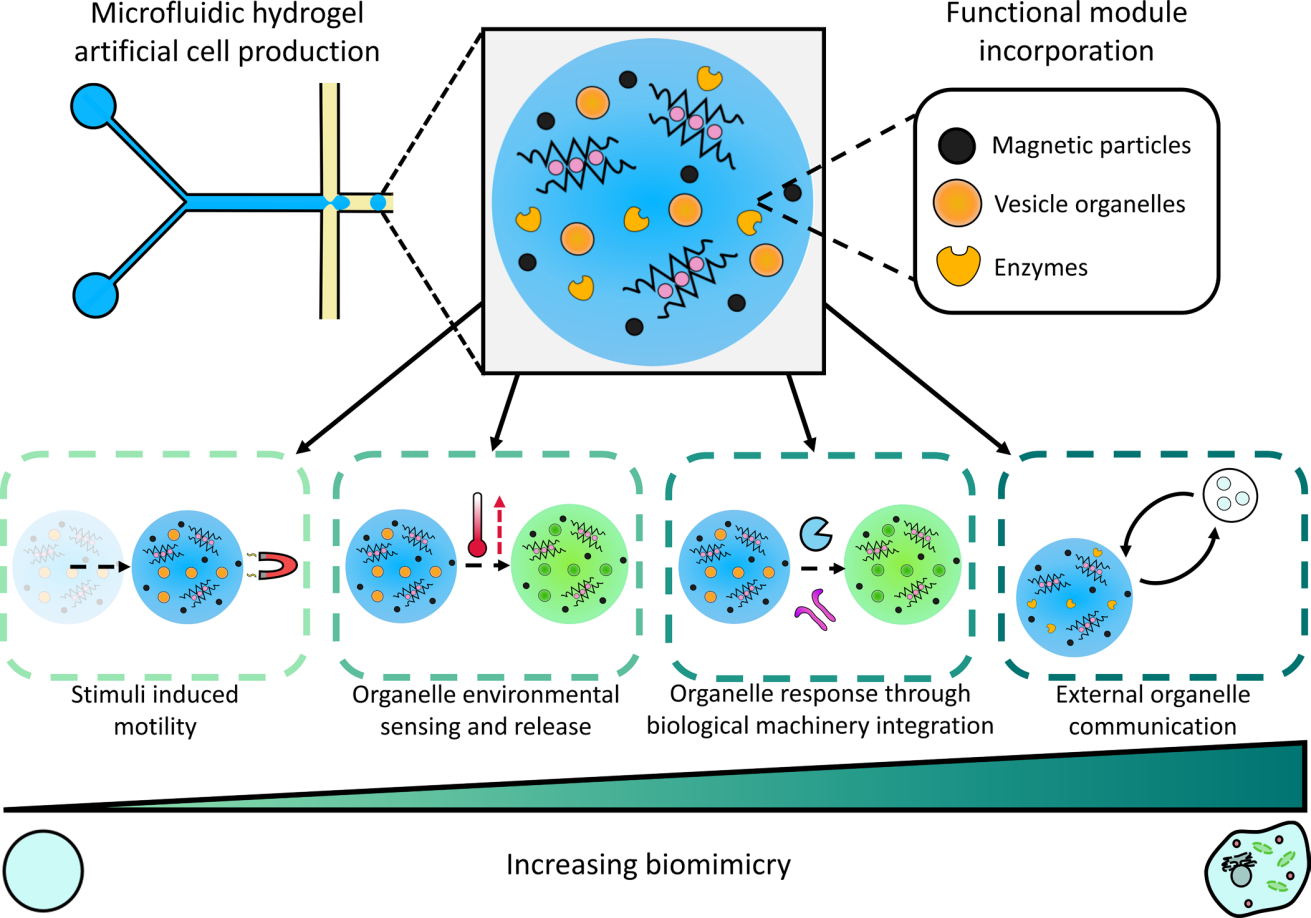
Design and function of the hydrogel artificial cells. Droplet microfluidics was used to create hydrogel-based artificial cells with a variety of different organelles and functional modules, including magnetic particles, vesicles, and enzymes, enabling the integration of a variety of biomimetic capabilities. In order of increasing biomimicry, these are stimulus-induced motility through the incorporated magnetic particles; cargo release from vesicle organelles in response to a change in environmental temperature; payload release from vesicle organelles through interactions with proteins and enzymatic biomarkers (biological machinery), and communication with external vesicle organelles through an enzyme embedded within the hydrogel artificial cell.

However, despite the clear potential of hydrogels as a chassis for artificial cells, they are underutilized. One reason for this is that the functionality of current hydrogel artificial cells are usually determined by the hydrogel material, which include polymeric materials such as DNA (33), polyacrylamide (34), poly (N-isopropylacrylamide) (35), agarose (36), and alginate (37). Specific materials are chosen to yield distinct functionalities, and multiple functionalities are seldom found in the materials used in hydrogel artificial cells. An issue which could be partially alleviated through the use of alternate hydrogel materials such as low-molecular weight gelators (38). However the ability to perform multiple functions is a key requirement to produce complex artificial cells that can mimic a range of varied cellular behavior. Here, we address this gap by developing a hydrogel artificial cell toolbox, which enables the design of functional microsystems with increasingly complex biomimetic features. This is achieved through using cell-seized hydrogel microparticles as artificial cell chassis in which are embedded a variety of biological and nonbiological modules (Fig. 1). The hydrogel frameworks are created through the gelation of alginate, a biocompatible polysaccharide (39) of a tailorable viscosity (40) that can readily contain both biological (41) and synthetic components (42), utilizing microfluidics (43, 44). We initially show that these hydrogel motifs can coencapsulate lipid vesicles, which act as organelles within the hydrogel chassis, and magnetic particles, which endow motility capabilities. We then display vesicle organelle activation and cargo release in response to both temperature changes and interactions with enzymatic biomarkers and membrane proteins. Moreover, using another hydrogel configuration involving encapsulated enzymes, we show the ability for our artificial cells to act as microreactors, as well as to communicate with neighboring cell-like compartments through activation of external vesicular systems. The versatility of our engineered microsystems—achieved through the range of modules encapsulated and biological operations replicated—will enable the creation of the next-generation artificial cells more closely mimicking cellular behaviors and processes within a hydrogel platform.

## Results

### Production and Characterization of Hydrogel Artificial Cells.

**Fig. 2.**
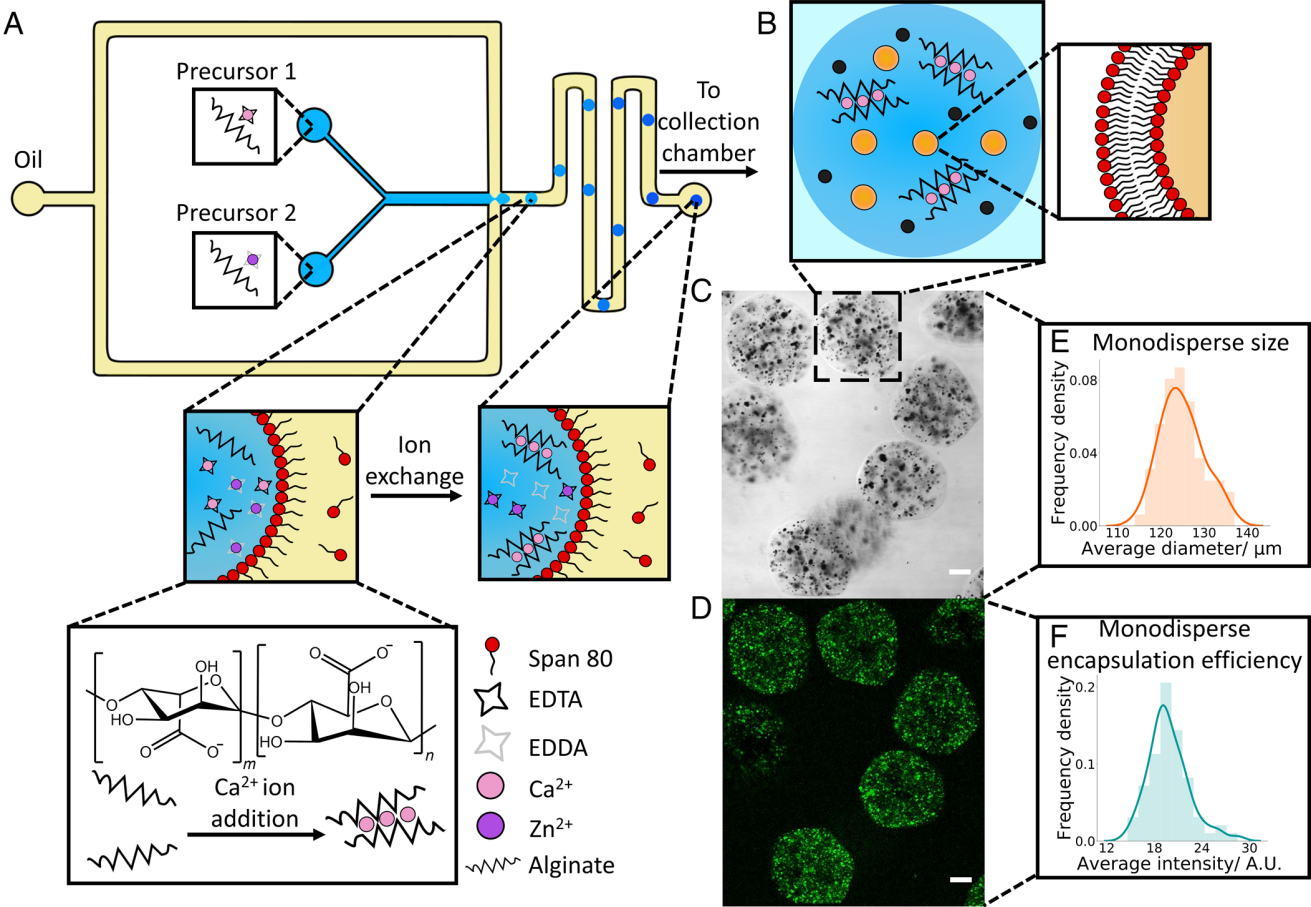
Production and characterization of hydrogel artificial cells. (*A*) Schematic illustrating the microfluidic chip design used to create the hydrogel artificial cells. Two aqueous precursor streams containing the two different alginate solutions meet an oil stream at a flow focusing junction where aqueous droplets stabilized by span 80 are generated. These aqueous droplets gelate through an ion exchange reaction where the Zn^2+^ ions displace Ca^2+^ from the EDTA, enabling the Ca^2+^ ions to crosslink the alginate chains together in an egg box structure producing the final hydrogel artificial cell chassis. The hydrogels are then collected in an external collection chamber. (*B*) Illustration of a typical hydrogel artificial cell within aqueous buffers showing embedded organelles including magnetic particles (black dots) and calcein containing vesicles (orange dots) within the hydrogel. The lipid bilayer of the vesicle organelles is shown in the enlarged diagram. (*C*) Brightfield confocal microscopy image of a population of hydrogel artificial cells containing magnetic particles and vesicle organelles. (*D*) Fluorescence confocal microscopy image of the same hydrogel population. The localization in fluorescent signal shows where the vesicles are embedded within the hydrogels. (*E*) Histogram demonstrating the average diameter of the hydrogels, the mean diameter was 125 µm with a polydispersity index of 0.0015. (*F*) Histogram showing the average fluorescence intensity of the hydrogels with a polydispersity index of 0.0166. The histograms were obtained from analyzing n = 70 hydrogels. Scale bar on all the microscopy images is 20 µm.

We prepared the hydrogel artificial cells, employing a microfluidic method based on the work of Håti et al. (45) (Fig. 2*A*). This method involved utilizing a polydimethylsiloxane (PDMS) chip (*SI Appendix*, Fig. S1) where two aqueous streams containing the hydrogel precursors (*SI Appendix*, Table S1) meet an oil stream at a flow focusing junction. At the flow focusing junction monodisperse aqueous droplets stabilized by span 80 were produced through ensuring the oil phase flow rate was larger than that of the combined aqueous precursor solutions. Within the aqueous droplets an ion exchange reaction between the calcium ethylenediaminetetraacetic acid (Ca-EDTA) and zinc ethylenediamine-N,N′-diacetic Acid (Zn-EDDA) complexes occurred whereby the Zn^2+^ ions displace the Ca^2+^ ions from the EDTA, thus enabling the Ca^2+^ ions to crosslink the alginate chains and produce a hydrogel within the aqueous droplet (*SI Appendix*, Fig. S2). Hydrogel formation was also confirmed through fluorescence recovery after photobleaching of the artificial cells (*SI Appendix*, Fig. S3) and a change in bulk mechanical properties (43). This ion exchange reaction was performed at pH 6.4 as it resulted in sufficiently fast gelation kinetics at a biocompatible pH without significant gelation occurring where the aqueous streams meet before the flow focusing junction (Movie S1). However, the pH range and kinetics of this gelation process can be tailored to suit a variety of purposes (45). We then resuspended the hydrogel artificial cells in an aqueous buffer to further characterize and perform experiments with.

The produced hydrogel artificial cell population which contained embedded magnetic particles (*SI Appendix*, Fig. S4) and 1-palmitoyl-2-oleoyl-glycero-3-phosphocholine (POPC) vesicle organelles (*SI Appendix*, Figs. S5 and S6) was characterized (Fig. 1*B*). The brightfield image (Fig. 2*C*) demonstrates the successful production of hydrogels and localization of the dark magnetic particles within the hydrogel structure. The presence of minimal magnetic particles within the external solution indicates that gelation occurs within the majority of the produced droplets. Furthermore, the range of sizes of the magnetic particle clusters occurred due to aggregation of the magnetic particles, a behavior also seen when the particles are suspended in bulk (*SI Appendix*, Fig. S4). Meanwhile the fluorescent image (Fig. 2*D*) exhibits a clear localization in fluorescence within the hydrogels, once again demonstrating encapsulation of POPC vesicles containing calcein. Moreover, as calcein is small (6.5 Å) (46) compared to the pore size of typical alginate hydrogels (a median value of ~10 nm) (47) any compromise of the vesicles membrane integrity both during the gelation process and within the hydrogel would lead to immediate dye leakage into the external buffer (*SI Appendix*, Fig. S7). The fluorescent signal being contained within the gels shows that the POPC vesicles are still retaining calcein within them and vesicle membrane integrity is not compromised by the hydrogel matrix. Finally, the clustering of the fluorescent signal within the hydrogels indicates that the vesicles sit within particular pores.

Throughout both the brightfield and fluorescence images, the magnetic particles and vesicles are spread throughout the hydrogels (Movies S2 and S3) demonstrating that the microfluidic gelation strategy enables an even distribution of encapsulated organelles. Often the structure of the hydrogel artificial cells is slightly nonspherical; this is occurring due to the close proximity of the aqueous droplets to each other within the exit chamber of the microfluidic device causing slight droplet deformation during the gelation process. However, to produce spherical hydrogels the exit chamber could be adjusted, or the aqueous flow rate could be reduced, thus enabling complete gelation within the microfluidic channels.

The population of the produced hydrogel artificial cells was additionally analyzed (Fig. 2 *E* and *F*), and it was seen that the hydrogels had a mean diameter of 125 µm and a poly dispersity index of 0.0015. The average fluorescence intensity of the encapsulated organelles additionally had a poly dispersity index of 0.0166. As values of <0.1 are typically considered monodisperse (48), microfluidics can be considered as a viable method for the production of monodisperse, hydrogel artificial cells with evenly encapsulated embedded organelles.

### Magnetically Induced Artificial Cell Motility.

**Fig. 3.**
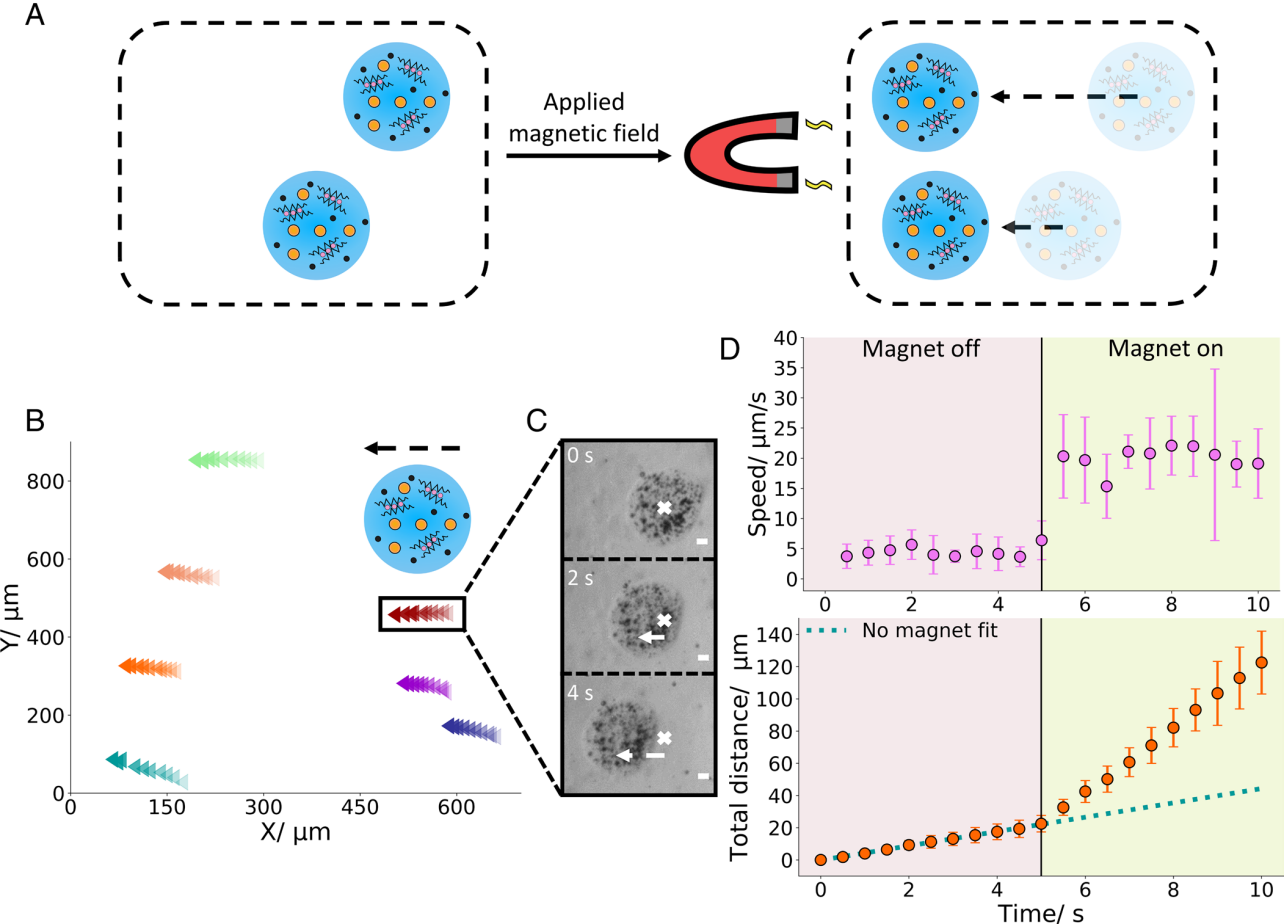
Motility of hydrogel artificial cells. (*A*) Diagram showing the motion of a population of hydrogel artificial cells to the right under an applied magnetic field. (*B*) Trajectories of seven different hydrogel artificial cells for 5 s. The direction of motion is indicated by the dashed arrow and the increasing opacity of the colored data points. (*C*) Brightfield images of a single hydrogel artificial cell with a magnetic field applied over the 4-s period, showing migration from the right (the cross marks the initial hydrogel position) to the left. (*D*) Speed and distance of hydrogel artificial cells before and after the application of a magnetic field. When the magnet is positioned close to the hydrogels after 5 s (shown by the change in background color), a significant change in speed is seen. Within the first 5 s on the distance graph a small change in distance is seen due to convection currents. After 5 s, a larger change in distance is observed due to magnetically induced motion. The blue dotted line illustrates the change in distance if no magnetic field was present after 5 s. The error bars indicate 1 σ (SD) from an n = 7 dataset. The scale bar on all the microscopy images is 20 µm.

After producing and characterizing the hydrogel artificial cells, we engineered them to move in response to an applied magnetic field (Fig. 3*A*). This motility could be induced by encapsulating magnetic particle organelles. When a magnet was placed approximately 1 mm away from a population of hydrogel artificial cell migration toward the placed magnet was observed (Fig. 3*B*) with the motion being driven by the encapsulated organelles (*SI Appendix*, Fig. S8). The observed hydrogel trajectories were nearly parallel to each other and directionally pointed toward the magnet indicating magnetically induced motion. This is further exemplified in Fig. 3*C* where the migration of a hydrogel artificial cell in the direction of the magnet is pronounced. Moreover, the encapsulated magnetic particle organelles do not migrate toward the magnet through the hydrogel (Movie S4) demonstrating that these organelles are held stably within the gel network.

To further illustrate the ability of the created artificial cells to respond to a magnetic field through the magnetic particles, we monitored the distance and speed over a 10-s interval with the magnet being introduced after 5 s (Fig. 3*D* and Movie S4). Within the first 5 s, a small change in distance and a small constant speed was observed in the tracked hydrogels, this distance and speed was predominantly occurring due to the convection currents in the sample chamber and was enhanced by the sample chamber possessing hydrophobic surfaces due to Rain-X treatment (49), thus reducing the interactions between the hydrogels and the sample chamber surfaces. Additionally, the distance was occurring in a random direction, indicating that no directional force was responsible for the gel movement at this point. After 5 s, once again, we placed the magnet approximately 1 mm away from the sample well. Upon placement a substantial increase in distance and a ~4.4-fold increase in speed was seen demonstrating that another force is now enabling more prominent hydrogel motion to be observed, the fitted dotted line in the distance graph shows the substantial difference between motion with and without the magnet. Furthermore, within Movie S4 directional movement can also clearly be seen upon magnet placement.

### Environmentally Activated Artificial Cell Content Release.

**Fig. 4.**
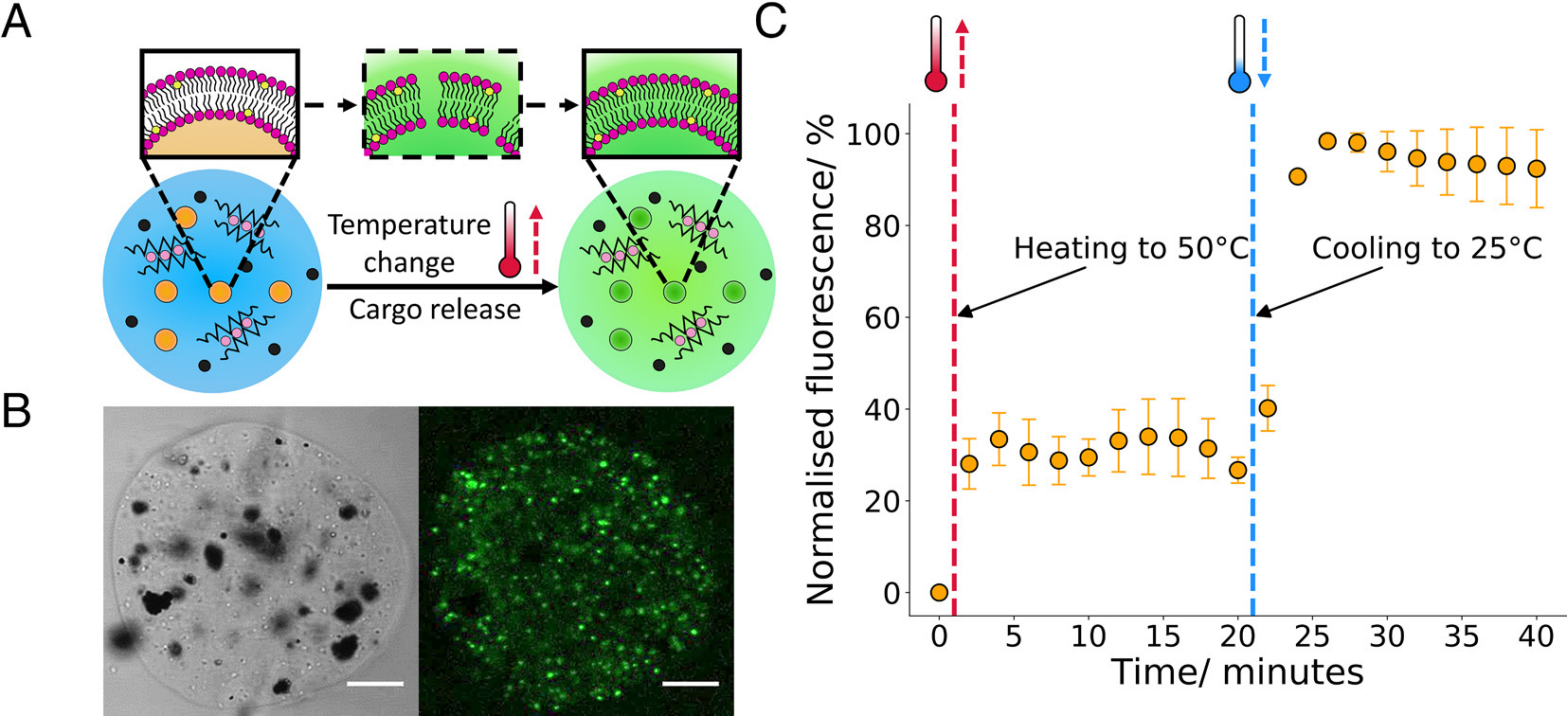
Environmentally activated hydrogel artificial cell organelles. (*A*) A schematic showing the temperature-dependent activation of the embedded vesicle organelles. Upon heating/cooling the vesicles through their phase transition temperature, the vesicle membrane exhibits defects (shown through the intermediate structure in the dotted box) which enables cargo release into the external environment from the vesicle compartment. (*B*) Brightfield and fluorescence confocal microscopy images of a hydrogel artificial cell with embedded thermoresponsive compartments, the localization in the fluorescence channel matches the smaller white aggregates seen on the brightfield channel indicating that these vesicles cluster within the hydrogel chassis. (*C*) Graph demonstrating calcein release from the embedded thermoresponsive vesicle organelles. With both heating and cooling through the vesicles phase transition, an increase in fluorescent signal is observed due to dye release through membrane defects which are present at the gel/fluid transition. The error bars indicate 1 σ from n = 3 hydrogel populations. The scale bar on all the microscopy images is 20 µm.

To further demonstrate the utility of the produced hydrogel artificial cells and their ability to respond to environmental cues, for instance a change in temperature, we incorporated thermoresponsive vesicle organelles of the composition 8:1, 1,2-dipalmitoyl-sn-glycero-3-phosphocholine (DPPC): Cholesterol into the hydrogel chassis instead of the POPC vesicles. This composition was chosen due to its success as a responsive component in other hydrogel systems (50) and a clear heating phase transition (*SI Appendix*, Fig. S9) between the gel and fluid phases at around 43 °C and a cooling phase transition between the fluid and gel phases at around 41 °C. The activation mechanism of such compartments is represented in Fig. 4*A* where a change in temperature can push the phospholipid membrane through a gel to fluid transition, leading to enhanced permeability (as shown in the dotted box) and cargo release (51).

As the vesicle organelle composition was altered, fluorescence and brightfield microscopy images were analyzed to ensure there was no significant alteration to the hydrogel artificial cell structure (Fig. 4*B*). The images illustrate that changing the composition of the vesicle compartments has a minimal impact on the hydrogel artificial cell structure. The fluorescence image again shows a localization in signal to regions where thermoresponsive vesicle organelles are present. Meanwhile, the brightfield image demonstrates the independent incorporation of the magnetic particles, allowing for two different functionalities to occur within the same artificial cell chassis.

After exhibiting that thermoresponsive vesicles can be inserted into hydrogel artificial cells as organelles in the same manner as POPC vesicles, we examined the ability of the thermoresponsive compartments to respond to temperature (Fig. 4*C*). Upon heating the hydrogel artificial cells from 25 °C to 50 °C at a rate of 20 °C min^−1^ and through the phase transition temperature, an immediate increase in fluorescent signal is observed due to the unquenching of encapsulated calcein. This indicates that calcein cargo release from the embedded vesicle organelles occurred through the produced membrane defects during the gel to fluid transition. After 20 min, the hydrogel artificial cells were cooled back to 25 °C at a rate of 20 °C min^−1^ and a another calcein release event was observed due to the vesicle membrane reverting back to the gel phase, in this reversion defects in the membrane are once again formed enabling calcein release to occur. In between the heating and cooling events no increase in fluorescence was observed as after the phase transition the reconfigured vesicle membrane is intact and thus impermeable to calcein. When compared to giant unilamellar vesicle (GUV) artificial cells with the equivalent organelles nested within (*SI Appendix*, Fig. S10), the hydrogel artificial cells can release their cargo directly into the external environment instead of into a vesicle lumen, which requires further activation to release into the environment. We therefore demonstrate that through using a hydrogel-based chassis, organelle interactivity with the external environment is more pronounced when compared to GUV-based systems.

### Triggering Artificial Cell Organelle Release Using Biological Machinery.

**Fig. 5.**
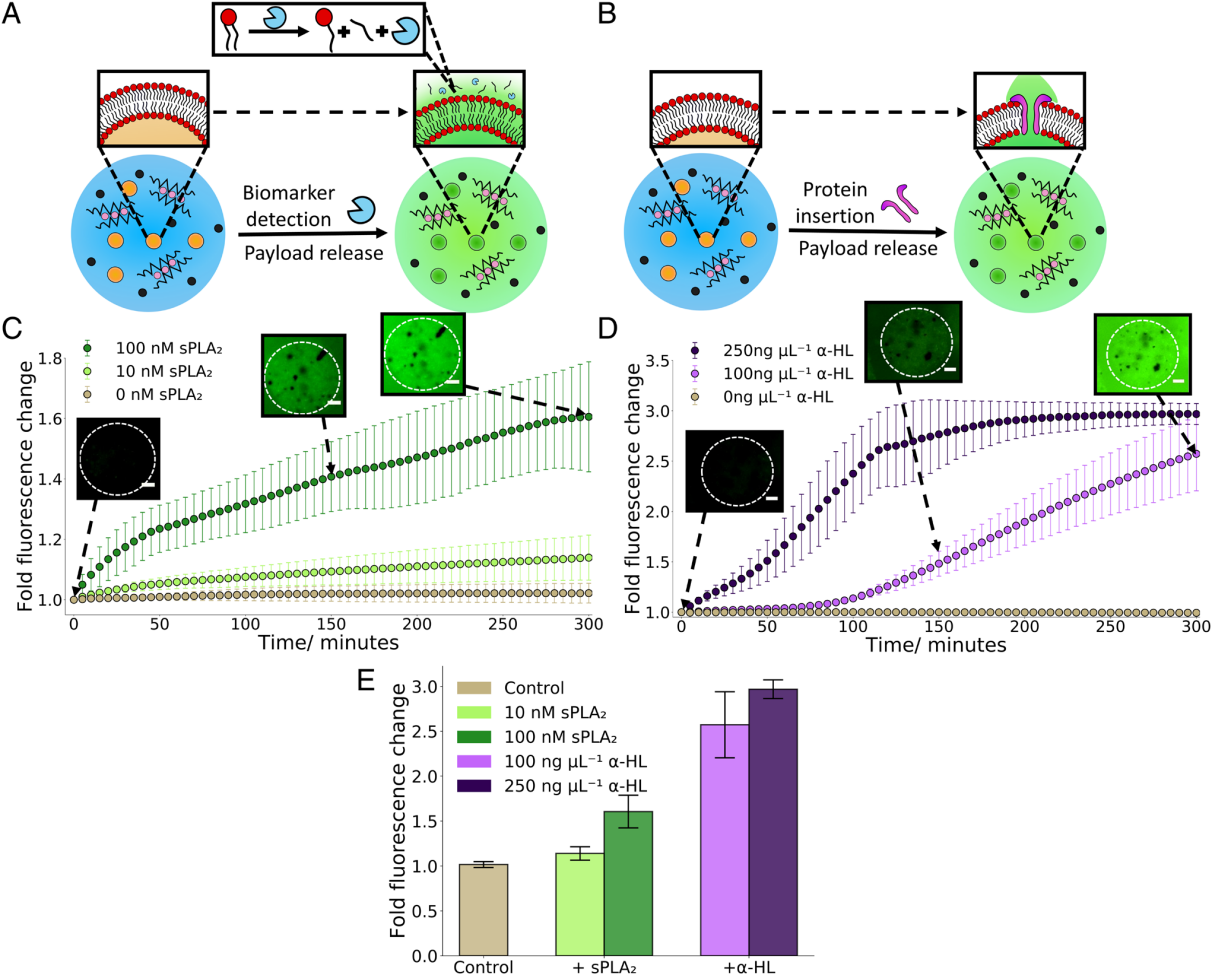
Triggering hydrogel artificial cell organelle content release with biological machinery. (*A*) A schematic demonstrating the activation of embedded calcein-filled vesicle organelles within the hydrogel chassis through the addition of the enzymatic biomarker sPLA_2_. After addition calcein cargo release and an increase in fluorescence will be observed due to the cleavage of lipid tails within the vesicle bilayer. (*B*) A 5-h timelapse graph showing how sPLA_2_ concentration impacts upon cargo release from the embedded vesicle organelles, a higher concentration of sPLA_2_ led to a faster release rate. There was negligible leakage without addition of sPLA_2_. The microscopy images at 0, 150, and 300 min demonstrate the increase in fluorescence associated with calcein release from the addition of 100 nM sPLA_2_ with the dotted circle showing the position of a hydrogel artificial cell. (*C*) An illustration showing the activation of embedded calcein-filled vesicle organelles within the hydrogel chassis through the addition of α-HL monomers. After addition calcein cargo release will be seen due to the presence of α-HL protein pores within the vesicle bilayer. (*D*) A 5-h timelapse graph showing how α-HL monomer concentration impacts upon cargo release from the embedded vesicles, a higher concentration of α-HL monomers led to a faster release rate. There was no leakage with no addition of α-HL. The microscopy images at 0, 150, and 300 min demonstrate the increase in fluorescence associated with calcein release from the addition of 100 ng µL^−1^ α-HL with the dotted circle showing the position of a hydrogel artificial cell. (*E*) A bar chart illustrating the difference in fluorescence change from the different biological machinery activation methods after 5 h. The addition of α-HL monomers led to a larger increase in fluorescence than the addition of sPLA_2_. The error bars on all the plots indicate 1 σ from n = 3 hydrogel populations. The scale bar on all the microscopy images is 20 µm.

After demonstrating the ability of the hydrogel artificial cells to respond to a magnetic field and release content in response to a change in environment, a synthetic biology approach was sought to further enhance the biomimicry of the system where cargo release from the embedded vesicle organelles was triggered using biological machinery. To demonstrate this, we encapsulated POPC vesicle organelles in the hydrogel chassis with the magnetic particles. POPC vesicles have a gel to fluid phase transition at −2 °C (52) and thus are in the fluid phase above this temperature; hence they will not release cargo in response to temperature. Instead varying concentrations of secretory phospholipase A2 (sPLA_2_), an enzyme overexpressed in tumor microenvironments (53) were added to the hydrogel artificial cells. The presence of the sPLA_2_ biomarker leads to the cleavage of phospholipid tail groups at the *sn-2* position, thus leading to the production of a fatty acid and lysophosphatidylcholine within the vesicle membrane (54). This production leads to the membrane permeability increasing (55) and the slow release of self-quenched calcein occurring from the vesicles and into the external environment causing an increase in fluorescence (Fig. 5*A*). As sPLA_2_ is ~14 kDa in size, it can readily diffuse into the hydrogel artificial cells and activate the vesicle organelles due to the enzyme being significantly smaller than the hydrogel pore size, as demonstrated in *SI Appendix*, Fig. S11 where a 70-kDa FITC-Dextran, a larger molecule readily diffuses into the hydrogel artificial cells.

Upon addition of the sPLA_2_ biomarker calcein cargo release from the embedded POPC vesicle organelles could be seen (Fig. 5*B*). Higher concentrations of sPLA_2_ (100 nM) are seen to lead to larger amount of cargo release and increases in fluorescence than lower sPLA_2_ concentrations (10 nM). This is due to higher concentrations of sPLA_2_ increasing the rate of cleavage of phospholipid tail groups and thus altering the membrane permeability more swiftly. Moreover, GUV-based artificial cells are significantly less stable than hydrogel-based artificial cells when exposed to this concentration of sPLA_2_ (*SI Appendix*, Fig. S12) thus showing the utility of using hydrogel artificial cells to mimic harsh environments when compared to predominantly lipid-based systems. In both concentrations of sPLA_2_, an initial quick increase is observed over the first 45 min before a more consistent increase in fluorescence. This initial increase is attributed to the first phospholipid cleavage events leading to a more dramatic change in permeability, predominately due to the vesicle compartments transitioning from a calcein impermeable structure to a partially permeable structure. The control demonstrates no passive calcein leakage without sPLA_2_ implying that the vesicle compartments are stable within the hydrogel chassis. The microscopy images further show that cargo release is occurring from the hydrogel artificial cell and into the surrounding environment through both an increase in fluorescence and delocalization of the signal from the hydrogel artificial cell to the entire environment.

After showing enzymatic biomarker-triggered organelle activation, an alternate biological mechanism was sought to activate the vesicle organelles to demonstrate that utilizing different biological machinery can give different organelle release profiles. For this, we added the protein α-Hemolysin (α-HL), a 33-kDa monomer that assembles into a heptameric transmembrane pore in vesicle bilayers (56, 57) to the hydrogel artificial cells. This leads to a release of self-quenched calcein from the vesicle organelle compartments and into the external environment through the protein pores produced within the vesicle membrane (Fig. 5*C*). Once again, the α-HL monomers were significantly smaller than the hydrogel pore size so could readily diffuse into the hydrogel artificial cell and activate the embedded organelles.

Upon the addition of α-HL to the hydrogel artificial cells, concentration-dependent calcein cargo release was seen from the vesicle organelles (Fig. 5*D*). The higher concentration of α-HL (250 ng µL^−1^) exhibits a fast increase followed by a plateau after ~ 200 min, this suggests at this concentration the entirety of the encapsulated calcein cargo has leaked into the external environment by this time point. Concurrently, the lower concentration of α-HL (100 ng µL^−1^) was still releasing calcein at 5 h. This is due to the lower α-HL concentration producing fewer protein pores in the vesicle bilayers thus slowing the diffusion speed of the calcein out of the vesicle organelles. Furthermore at 100 ng µL^−1^ of α-HL a lagged release curve is observed. This occurs as α-HL requires 7 monomers to insert into a vesicle bilayer in order to form a protein pore to release the contained calcein, at a lower concentration of α-HL fewer insertion events will occur, thus increasing the time required for α-HL pores to form and enable calcein release. The microscopy images again illustrate that calcein cargo release is occurring from the hydrogel artificial cell and into the surrounding environment through both an increase in fluorescence and the delocalization of the signal from the hydrogel artificial cell to the entire environment.

As we utilized different biological machinery to activate the vesicle organelles, the choice of activation strategy was readily compared in Fig. 5*E*. After 5 h it is evident that using α-HL proteins as an activator led to a twofold increase in calcein release from the vesicle organelles than using the sPLA_2_ biomarker. This occurs due to α-HL creating a transmembrane pore in the vesicle compartments thus enabling a channel linking the vesicle interior to the external environment. In comparison sPLA_2_ only cleaves phospholipids, which creates minor defects in the bilayer and thus no direct link to the external environment (*SI Appendix*, Fig. S13). The difference between the two biological machinery activation mechanisms illustrates that the vesicle compartment release rate can be tuned by both the machinery used and the concentration.

### Hydrogel Artificial Cells as External Organelle Communication Tools.

**Fig. 6.**
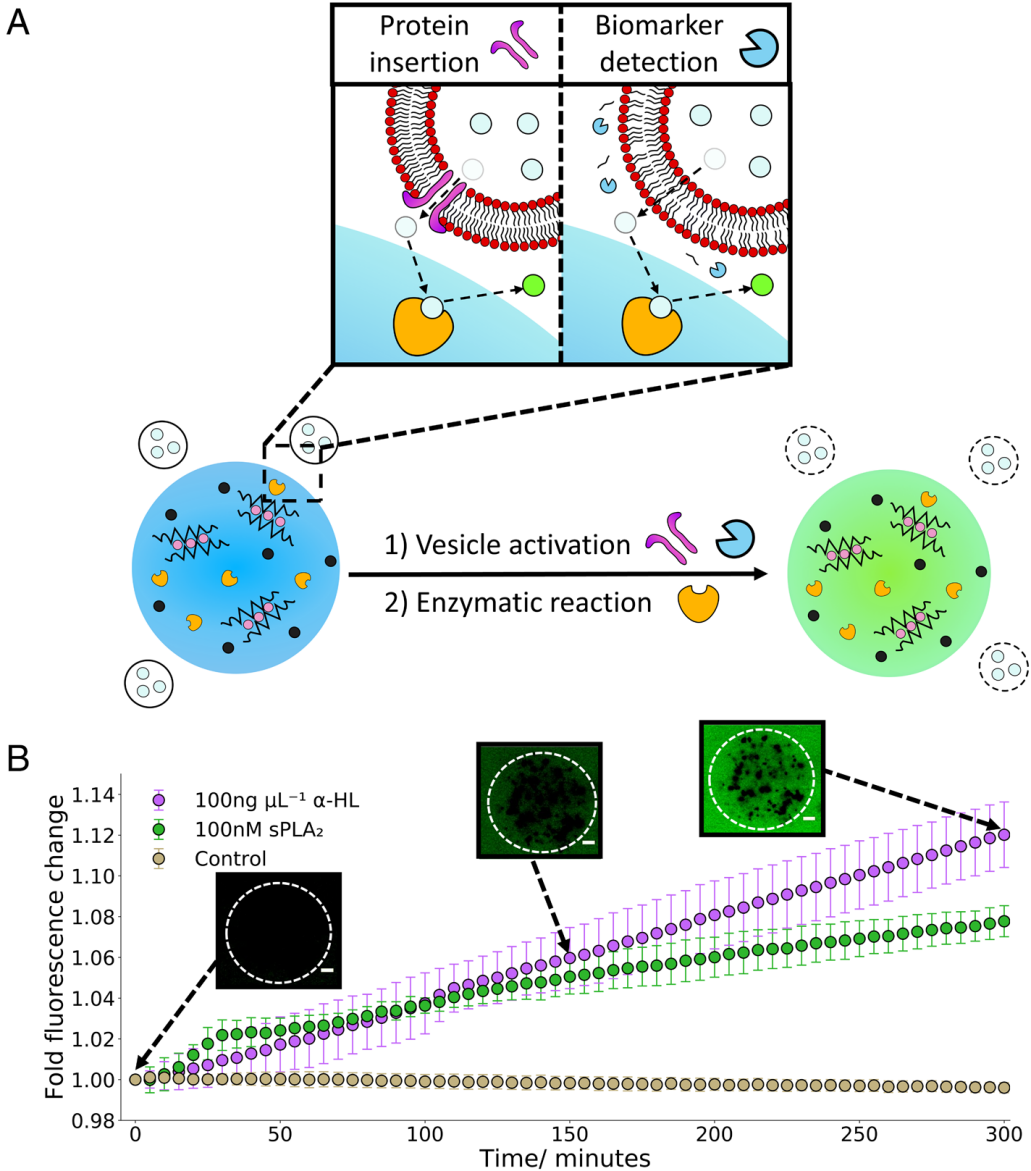
Hydrogel artificial cells as external organelle communication tools. (*A*) A drawing indicating hydrogel artificial cells with embedded β-Galactosidase interacting with a population of surrounding vesicle organelles containing FDG. When the surrounding vesicles are activated using either sPLA_2_ or α-HL, release of FDG occurs (the light blue circles), the FDG then diffuses into the hydrogel artificial cells where it is converted into fluorescein (the bright green circles), this process is shown in the box. This leads to an increase in fluorescence of the entire system. (*B*) A 5-h timelapse chart illustrating the activation of the external vesicle organelles using sPLA_2_ and α-HL and the conversion of FDG to fluorescein using the embedded β-Galactosidase in the hydrogels. After 5 h, the α-HL triggered system has led to a faster conversion of FDG to fluorescein with the hydrogels than the sPLA_2_. The microscopy images at 0, 150, and 300 min demonstrate the increase in fluorescent signal associated with the conversion of released FDG to fluorescein from the addition of 100 ng µL^−1^ α-HL. The error bars indicate 1 σ from n = 3 hydrogel populations. The scale bar on all the microscopy images is 20 µm.

After illustrating that our hydrogel artificial cells can contain a variety of organelles that can respond to physical and chemical triggers, their ability to contain biological components and to communicate with external organelles was probed to further enhance the biomimetic nature of the artificial cells. We placed the enzyme β-Galactosidase (β-Gal) into the hydrogels, replacing the vesicle organelles, both due to the large size of the enzyme at 465 kDa (58) and previous success at encapsulating this enzyme within hydrogel systems (59). The hydrogel artificial cells were then placed in a buffer that additionally contained a population of POPC vesicles containing fluorescein di-β-D-galactopyranoside (FDG) molecules (Fig. 6*A*) which acted as external organelles for the hydrogel artificial cells to communicate with. Upon triggered leakage of the external vesicles (using sPLA_2_ and α-HL as described above), the FDG freely diffuses into the hydrogel artificial cells where it is converted by the entrapped β-Gal into fluorescein, a fluorescent product (60). The fluorescein then diffused into the external environment leading to an increase in observed fluorescent signal throughout the sample. Through activating the external vesicle organelles, the hydrogel artificial cells are communicating through receiving a signal in form of FDG before sending the converted fluorescein back into the environment.

The ability of the hydrogel artificial cells to communicate with the external organelles is demonstrated within Fig. 6*B*. Upon adding either form of biological machinery (sPLA_2_ or α-HL) to the external vesicle organelles, an increase in fluorescent signal was observed, unlike the control, indicating the successful release of FDG and conversion to fluorescein inside the hydrogel artificial cells. A short delay of ~15 min was observed before an increase in fluorescent signal in the activated samples. This is due to the requirement of the FDG to be released, diffuse to the enzymatic sites within the hydrogels and be converted to fluorescein before a fluorescence increase is observed, a process that is not instantaneous. As seen within Fig. 5 *B* and *D*, the addition of sPLA_2_ caused an initial quick release step followed by a slower release while the α-HL presented a more controlled release. Once more the α-HL led to greater fluorescent signal change than the sPLA_2_. The microscopy images further demonstrate an increase in fluorescent signal over the 5-h observation period, the fluorescence however is even across the whole image due to the lack of embedded fluorescent structures in the hydrogels and the produced fluorescein freely diffusing through the environment.

To confirm the enzymatic reaction was occurring within the hydrogel artificial cells and not in the surrounding buffer, after 1 d at room temperature the hydrogels were resuspended into fresh buffer containing FDG (*SI Appendix*, Fig. S14). Fluorescein conversion was once again seen confirming that β-Gal is still present and active in the hydrogel system and had not completely leaked out into the surrounding buffer over this timeframe.

## Discussion

Within this work, we demonstrate the inclusion of a myriad of organelles into hydrogel artificial cells, where the organelles act as functional modules with a variety of different applications and properties to mimic cellular functions. One of these organelles are magnetic particles that endow functional motility and can be activated on demand, a key step toward the recreation of biomimetic structures. Furthermore, the magnetic particles can also be used for protein purification and consist of a solid bead to which the nickel is attached to (61). As a consequence, encapsulation of similar solid organelles will be facile and enable the functional repertoire of the hydrogel artificial cells to be readily expanded, for instance through utilizing solid particles as biosensors (62).

Another functional organelle we incorporated into the hydrogel artificial cells are thermosensitive vesicles. These vesicles can be activated by the environment in an independent manner to the magnetic motion. Hence different unrelated stimuli can be applied to activate different organelles either individually or simultaneously. This paves the way toward orthogonally releasing different cargo from different compartments or enabling different compartments to be activated in a cascade manner. Such behavior is required for the engineering of more complex microsystems.

Moreover, within the produced hydrogel artificial cells, we can alter the functional organelles in an entirely independent manner to the hydrogel chassis. For instance, we altered the vesicle organelle composition to provide compartment activation with biologically relevant machinery (tumor-expressed enzymes and proteins). This paves the way for hydrogel artificial cells to interact with more complex biological systems, be used as cancer-targeting therapeutics or toward the production of biohybrid systems within a hydrogel chassis (9). This demonstrates a level of control seen in complex biosystems that can be readily integrated into these structures. We also show that the hydrogel artificial cells more readily tolerate harsher conditions when compared to lipid-based artificial cells, enabling them to be cell mimics across a greater functional space.

Furthermore, we additionally utilize the hydrogel artificial cells as tools to dynamically communicate with external functional moieties within the external environment, not just as a chassis to hold functional organelles. This is another key characteristic of living systems that the hydrogel artificial cells are replicating and in a different manner to traditional impermeable vesicle based artificial cells due to the hydrogels being semipermeable, thus their structure further provides a selective barrier, enabling small molecules to readily communicate with internal hydrogel artificial cell organelles but preventing larger moieties from doing so. Additionally, this paves the way for hydrogel artificial cells to communicate with living systems such as bacteria (63).

In conclusion we have developed a toolkit for the microfluidic assembly of monodisperse micron scale hydrogel-based artificial cells, which contain multiple interchangeable organelles of both a biological and synthetic nature acting as operative modules. These functional organelles enable the recreation of increasingly biomimetic behavior. This includes magnetically induced motility, controlled cargo release in response to external environmental conditions and biological machinery integration, and communication with external vesicle organelles. The ease of organelle interchangeability also allows for “plug and play” functionality, which enables the facile replication of complex cellular behaviors. Our platforms will enable the design of classes of artificial cells with increasingly complex behaviors, for instance through the incorporation of alternative functional modules such as genetic componentry, protein expression machinery and chloroplasts, as well as the production of biohybrid hydrogel artificial cells with embedded bacteria. Moreover through the fusion of this technology with existing 3D alginate printing platforms (64), tissue-like materials with compartmentalized modules could be generated. Alternatively through the utilization of more sophisticated microfluidic approaches and 3D printing for rapid prototyping of different device designs, the ability to further compartmentalize the hydrogel artificial cells and create more elaborate and biomimetic architectures could be achieved (65, 66). Furthermore due to the alginate hydrogel artificial cell chassis being biocomptabile (39), our structures could be readily used as next-generation microsystems with applications as diverse as drug delivery, biosensing, and bioremediation.

## Materials and Methods

### Materials.

The lipids POPC, 1,2-dioleoyl-sn-glycero-3-phosphocholine (DOPC) and 1,2-dipalmitoyl-sn-glycero-3-phosphocholine (DPPC) were purchased from Avanti Polar Lipids (Alabaster, AL) as powders and used without further purification. Sodium alginate was obtained from Sigma Aldrich (Gillingham, UK) and used without further purification. The MagneHis^TM^ Ni-Particles were purchased from Promega (Southampton, UK). The secretory phospholipase A2 (sPLA2) from honeybee venom (Apis mellifera), α-Hemolysin from *Staphylococcus aureus* (α-HL), β-Galactosidase from *Escherichia coli and* fluorescein di-β-D-galactopyranoside (FDG) were all obtained from Sigma-Aldrich (Gllingham, UK). PDMS Sylgard 184 elastomer kits were purchased from Dow Corning (Michigan, USA). Rhodamine B-labeled alginate was purchased from HAworks (New Jersey, USA). EDDA was purchased from Tokyo chemical industry. The remaining reagents which include sucrose, glucose, Bovine Serum Albumin (BSA), cholesterol, HEPES buffer, potassium chloride, calcium chloride, zinc acetate, ethylenediaminetetraacetic acid (EDTA), sorbitan monooleate (Span 80), mineral oil, sephradex G-50, and 70-kDa-Fluorescein isothiocyanate-dextran were all purchased from Sigma Aldrich (Gillingham, UK) unless specified.

### Vesicle Production.

Lipid films were produced by weighing out appropriate amounts of lipid and dissolving them in chloroform. For the POPC vesicles, 10 mg of POPC was weighed out. For the thermal vesicles which consists of the composition 8:1 (mol: mol) DPPC: Cholesterol, 9.38 mg and 0.62 mg, respectively, of the lipids were weighed out. The lipid in chloroform solutions were then mixed for 1 min before the chloroform was gently evaporated under a stream of N_2_. The resultant films were then left under vacuum overnight to remove the residual chloroform. Films were then rehydrated with 100 mM KCl, 100 mM HEPES (pH 7.4), and either 50 mM calcein or 0.75 mM FDG. The final concentration of the lipid solutions was 20 mg mL^−1^. The lipid solutions were then freeze–thawed five times for the calcein encapsulated vesicles or 10 times for the FDG encapsulated vesicles. This was done through flash freezing the vesicle suspension in liquid N_2_ before thawing the sample by heating to 50 °C and vortexing for 30 s. The vesicles were then extruded through 0.2-μm polycarbonate membranes 21 times to produce a vesicle population of ~180 nm in size (*SI Appendix*, Fig. S5). Vesicles were then passed a size exclusion column to remove unencapsulated calcein or FDG. This utilized a Sephadex G-50 column containing an eluent of 0.5 M sucrose and 100 mM KCl. Then, 200 μL of vesicles was added to the column, and purified vesicles were collected in fractions of 300 μL. All vesicles were used within 2 d of production to minimize cargo leakage.

The vesicles used to make the nested giant unilamellar vesicle system were rehydrated with 100 mM KCl, 100 mM HEPES (pH 7.4), 20 mM CaCl_2_ and 50 mM calcein and collected with a column eluent of 0.5 M sucrose, 100 mM HEPES, 100 mM KCl, 20 mM CaCl2 pH 7.4.

### Microfluidic Device Manufacture.

The silicon master wafers (Inseto) were first created by depositing a photoresist (SU-8 3050, Kayaku Advanced Materials, MA, USA) of 100-µm depth using a spin coater. The wafers were then baked before UV light exposure (365 nm, 300 mJ cm^−2^) through an acetate photomask (Micro Lithography services, UK) containing the device design. After a postexposure bake, the unexposed features were removed using propylene glycol monomethyl ether acetate developer and rinsed with Isopropyl alcohol. The patterned wafers were finally silanized with trichloro(1H, 1H, 2H, 2H-perfluorooctyl) silane under vacuum overnight.

The patterned wafers then had degassed PDMS (10:1 Elastomer: Curing agent) poured onto them and were left to cure for at least 3 h at 60 °C. The patterned PDMS was then removed from the underlying wafer, 1.5-mm holes were punched for the inlet and outlet ports before being irreversibly bonded to a glass slide in order to seal the microfluidic channels. This was accomplished by exposing both the glass slide and patterned side of the PDMS to plasma (Harrick Plasma, NY, USA) for 1 min before contacting the surfaces together. The devices were then left overnight before use to ensure complete bonding between the glass slide and PDMS.

### Hydrogel Production.

Stock solutions of 2 wt% alginate, 0.5 M sucrose, 84 mM Ca-EDTA or 84 mM Zn-EDDA and 40 mM HEPES (pH 6.4) were made up. These were combined with the purified vesicles (diluted to 40% of the purified vesicle solution) and magnetic particles (diluted 10-fold from the purchased stock) to give the final aqueous solutions containing 1 wt% alginate, 0.45 M sucrose, 40 mM KCl, 42 mM Ca-EDTA or 42 mM Zn-EDDA, 20 mM HEPES (pH 6.4), 10 mg mL^−1^ magnetic particles and purified vesicles. For the enzymatic reaction experiments, 5 units mL^−1^ of β-Galactosidase was added to both aqueous phases instead of the vesicles thus leading to aqueous solutions containing 1 wt% alginate, 0.45 M sucrose, 42 mM Ca-EDTA or 42 mM Zn-EDDA, 20 mM HEPES (pH 6.4) and 10 mg mL^−1^ magnetic particles. The full list of compositions used for each experiment can be seen in *SI Appendix*, Table S1. The oil phase was prepared through dissolving 5 wt% Span 80 into mineral oil. The addition of Span 80 into the continuous oil phase increased the stability of the produced aqueous droplets and consequently reduced coalescence and formation of larger than intended alginate hydrogels.

The aqueous and oil phases were then added into the appropriate inlets of the PDMS microfluidic device (As demonstrated in *SI Appendix*, Fig. S1). Polyethylene tubing (Kinesis, UK) was used to deliver solutions both to and from the microfluidic chip and a pressure pump (Elveflow, Paris, France) was utilized to control the flow rates of the aqueous and oil phases. The aqueous flow rates were always identical to ensure an equal distribution of both aqueous phases and the oil phase flow rate was always larger than that of the aqueous flow rates to ensure droplet formation occurred. The size of the aqueous droplets was dictated by the channel geometries thus smaller hydrogels could be readily produced if required by shrinking the size of the channels. With the device depicted in *SI Appendix*, Fig. S1, hydrogels with an average size of 125 µm were generated.

The produced hydrogels were collected by outlet tubing from the microfluidic device connecting to an Eppendorf tube. The collected hydrogels were then centrifuged for 5 min at 9,000 × g to produce a pellet. The oil phase supernatant was then removed before the pellet was resuspended in sucrose buffer (0.5 M sucrose, 100 mM HEPES, 100 mM KCl, 20 mM CaCl_2_ pH 7.4). The additional calcium in this buffer was to prevent any unwanted hydrogel degradation from occurring.

### Nested Giant Unilamellar Vesicle Production.

The nested giant unilamellar vesicles were made through hydrating a 2-mg film of DOPC in mineral oil to create a 2 mg mL^−1^ lipid in oil solution. This was sonicated for 30 min at 40 °C to ensure the lipids were fully dissolved. A column was then prepared in an Eppendorf containing 200 µL of 0.5 M glucose, 100 mM HEPES, 100 mM KCl, 20 mM CaCl_2_ pH 7.4 with 50 µL of lipid in oil deposited on top. This was left for 30 min to enable efficient monolayer assembly at the oil/water interface. An emulsion was then created by adding 20 µL of the purified vesicle solution to 200 µL of the lipid in oil solution and mixing through pipetting up and down 20 times. The emulsion was layered on top of the created column and centrifuged for 10 min at 9,000 x g to produce a pellet containing the nested giant unilamellar vesicles. The pellet was resuspended in 0.5 M glucose, 100 mM HEPES, 100 mM KCl, 20 mM CaCl_2_ pH 7.4 and washed once more through using the same centrifugation cycle before being visualised under a microscope.

### Microfluidic Microscopy.

A NikonTE2000-E microscope with a Ximea MQ013MG-E2 camera and a 1× objective was used to record the brightfield timelapse videos for the microfluidic production of hydrogels.

### Fluorescence Spectroscopy of Unencapsulated Vesicles.

A Cary Eclipse fluorescence spectrophotometer (Agilent, Santa Clara, USA) was used to observe calcein release by measuring the fluorescent emission of calcein at 514 nm (excitation at 494 nm). Then, 200 µL wells on a black 96-well plate were filled with vesicles in sucrose buffer at a 1:10 ratio. Baseline readings were obtained for 10 min before a 3% (v/v%) Triton X-100 solution was added to enable full vesicle lysis. After the addition of Triton X-100 readings were taken for another 20 min to confirm full lysis had occurred. The fluorescent readings for the vesicles were normalized by applying the below equation (Eq. **1**) where $F_{0}$ was the initial fluorescent reading, $F_{t}$ was the fluorescence at a given time point, and $F_{\max}$ was the maximum fluorescent reading obtained.

[1] $$\begin{array}{c} \text{Normalized}\;\text{fluorescence}\left(\%\right)=\left(\frac{F_{t}-F_{0}}{F_{\max}-F_{0}}\right)\times 100. \end{array}$$

### Dynamic Light Scattering of Lipid Vesicles.

A Malvern Zetasizer Ultra instrument (Malvern, UK) with a 632.8-nm HeNe gas monochromatic laser was used to size the vesicles using backscattered light. Scattered light was detected at an angle of 173${}^{\circ }$ from the transmitted beam in order to minimise the collection of unwanted reflection. Vesicle samples were measured by diluting the purified vesicles in a 1:10 ratio in sucrose buffer (0.5 M sucrose, 100 mM HEPES, 100 mM KCl, 20 mM CaCl_2_ pH 7.4).

### Confocal Microscopy.

A Leica TCS SP5 confocal microscope was used with a 20× or 10× objective and an 85- or 70.7-µm pinhole, respectively. The samples containing calcein-filled vesicles or FITC-Dextran were excited at a wavelength of 488 nm, and the emission spectra between 500 and 535 nm was monitored. The samples were imaged in a PDMS well upon a coverslip. Another coverslip was then placed on top to seal the sample chamber. The size distribution of the hydrogels was then obtained through measuring a population using the ImageJ software. Likewise, the average intensity distribution of the hydrogels was calculated through measuring the fluorescence intensity of the in-plane hydrogels using ImageJ (NIH, USA). The polydispersity index (PDI) of the size and intensity distributions was calculated using Eq. **2** where σ is the SD of the distribution and d is the mean (67).

[2] $$\text{PDI}={\left(\frac{\sigma }{d}\right)}^{2}.$$

For the fluorescence recovery after photobleaching experiments and the calcein permeation control, a Leica stellaris 8 was used with a 10×/0.4 NA objective and a 532-nm or a 480-nm excitation laser, respectively. The respective recorded emission spectra had maximum values at 568 and 515 nm, respectively. The photobleaching data was then normalized using the following equation where $F_{\max}$ is the maximum fluorescence signal and $F_{t}$ is the fluorescence at a given time point.

[3] $$\text{Normalized}\;\text{fluorescence}=\frac{F_{t}}{F_{\max}}.$$

### Magnetic Movement Experiments.

To produce the sample wells for the magnetic movement experiments a laser cutter (VLS 2.30 Universal Laser Systems, Austria) was used to cut wells in 2 mm hard continuous poly(methyl methacrylate) (Clarex 001, Weatherall Equipment and Instruments Ltd, UK) with double-sided acrylic adhesive. The wells were then attached to a glass slide before being treated with Rain-X to limit gel adherence to the glass surface. For all experiments performed, the distance between the well for the magnet and the sample well was approximately 1 mm. For recording the brightfield magnetic movement videos, a Nikon TE2000-U inverted microscope with a Ximea MQ013MG-E2 camera and 4× objective was used. To induce magnetic motion a QuickPick™ 1-M Magnetic tool containing a 3-mm neodymium radial magnet was placed in the magnet well on the microscope stage. The recorded videos were then analyzed using the manual tracking tool on ImageJ by selecting the center of the gels at 0.5-s intervals to extract the X and Y coordinates. These coordinates were used to plot the hydrogel trajectories and used to calculate the total distance of the hydrogels using Eq. **4**. Within this equation X and Y are the x and y coordinates of the hydrogel, n is the number of timesteps analyzed and i is the current timestep.

[4] $$\begin{array}{c} \text{Total}\;\text{distance}=\sum_{i=1}^{n}\sqrt{({X_{i+1}-X_{i})}^{2}+({Y_{i+1}-Y_{i})}^{2}}. \end{array}$$

Eq. **5** was used to calculate the speed of the hydrogels through the difference in distance between two distance data points ( Δs ) and dividing by the time interval between these points ( Δt).

[5] $$\text{Speed}=\frac{\Delta s}{\Delta t}.$$

### Differential Scanning Calorimetry (DSC).

A known weight of a 70 wt% hydrated 8:1 DPPC: cholesterol mixture was placed in hermetically sealed aluminum DSC pan purchased from PerkinElmer. Before being placed in the pan the sample was subjected to 5 freeze–thaw cycles. A PerkinElmer Diamond DSC was then used to obtain a heating thermogram of the sample.

### Timelapse Microscopy.

A Nikon eclipse Ti2-U inverted microscope with a CoolLED pE-300^white^ and a Nikon DS-Qi2 camera was used to image the hydrogels, giant unilamellar vesicles, magnetic particles, hydrogel emulsions, and perform the timelapse experiments. For the hydrogels containing embedded vesicles activated by sPLA_2_ and α-HL, the sPLA_2_ and α-HL were added into buffer solution. For the enzymatic reactions, the buffer solution additionally contained a population of purified FDG vesicles diluted twofold. These solutions were imaged using PDMS wells on a coverslip, another coverslip was placed on top to seal the sample chambers. In the case of giant unilamellar vesicles, the coverslips were functionalized with a 1 wt% BSA solution through coating the slip before drying in an oven. Fluorescence imaging was done with a GFP filter cube to image the calcein and fluorescein dyes. In the case of the hydrogel emulsion Rain-X was applied to the bottom coverslip to prevent aqueous droplet adherence. After recording the timelapses, Eq. **6** was used to calculate the fold fluorescence change. In the equation, $F_{t}$ was the fluorescence intensity at a given timepoint and $F_{0}$ was the initial fluorescent intensity.

[6] $$\text{Fold}\;\text{fluorescence}\;\text{change}=\frac{F_{t}}{F_{0}}.$$

For the heating timelapse experiments a Linkam PE120 Peltier heating stage with a EHEIM professional 4+ water filter unit was placed on the microscope. The hydrogel samples were heated and cooled between 50 °C and 25 °C at a rate of 20 °C min^−1^. The obtained timelapses were then normalized using Eq. **1** where $F_{0}$ was the initial fluorescent reading, $F_{t}$ was the fluorescence at a given time point and $F_{\max}$ was the maximum fluorescent reading obtained.

## Supplementary Material

Appendix 01 (PDF)

Movie S1. A timelapse demonstrating hydrogel production using a microfluidic device.

Movie S2. Brightfield Z stack showing magnetic particle localisation within a hydrogel.

Movie S3. Fluorescent Z stack showing POPC vesicle localisation within a hydrogel.

Movie S4. Timelapse of hydrogel movement before and after magnet application.

Movie S5. Timelapse of calcein release from a hydrogel using 100 ngμL^-1^ of α-Hemolysin.

Movie S6. Timelapse of conversion of FDG to Fluorescein using β-Galactosidase embedded in a hydrogel. The FDG is released from surrounding vesicles with 100 ngμL^-1^ α-Hemolysin.

## Data Availability

All study data are included in the article and/or supporting information.
