# Supplementary material for: Biomimetic behaviors in hydrogel artificial cells through embedded organelles
Source: Proc Natl Acad Sci U S A. 2023 Aug 21;120(35):e2307772120. doi: 10.1073/pnas.2307772120 (PMC10466294; doi:10.1073/pnas.2307772120)
Supplement: Supplementary file 1 — Appendix 01 (PDF) [file pnas.2307772120.sapp.pdf]

PNAS

Supporting Information for Biomimetic behaviours in hydrogel artificial cells through embedded organelles

Matthew E. Allen[1,2,3], James W. Hindley[1,3], Nina O'Toole[2,3], Hannah S. Cooke[1,2,3], Claudia Contini[2,3], Robert V. Law[1,3], Oscar Ces[1,3], Yuval Elani[2,3]*

[1]Department of Chemistry, Imperial College London, Molecular Sciences Research Hub, London, W12 0BZ, UK

[2]Department of Chemical Engineering, Imperial College London, South Kensington, London SW7 2AZ, UK

[3]FabriCELL, Imperial College London, Molecular Sciences Research Hub, London, W12 0BZ, UK

*Yuval Elani

Email: y.elani@imperial.ac.uk

This file includes:

Table S1
Figures S1 to S14
Legends for Movies S1 to S6

Other supporting materials for this manuscript include the following:

Movies S1 to S6

Figure	Description	Precursor 1 composition	Precursor 2 composition
Figures 2, 3 and 5 and Figures S2, S8, S11	Magnetic motility and enzymatic biomarker/ protein triggered cargo release	1 wt% alginate, 0.45 M sucrose, 40 mM KCl, 42 mM Ca-EDTA, 20 mM HEPES (pH 6.4),10 mg/ml MagneHis^{TM} Ni-Particles, 200 nm calcein containing POPC vesicles	1 wt% alginate, 0.45 M sucrose, 40 mM KCl, 42 mM Zn-EDDA, 20 mM HEPES (pH 6.4), 10 mg/ml MagneHis^{TM} Ni-Particles, 200 nm calcein containing POPC vesicles
Figures 4 and S10	Environmentally (thermally) triggered content release	1 wt% alginate, 0.45 M sucrose, 40 mM KCl, 42 mM Ca-EDTA, 20 mM HEPES (pH 6.4),10 mg/ml MagneHis^{TM} Ni-Particles, 200 nm calcein containing thermal vesicles	1 wt% alginate, 0.45 M sucrose, 40 mM KCl, 42 mM Zn-EDDA, 20 mM HEPES (pH 6.4), 10 mg/ml MagneHis^{TM} Ni-Particles, 200 nm calcein containing thermal vesicles
Figures 6 and S14	Enzymatic biomarker/ protein triggered enzymatic reaction	1 wt% alginate, 0.45 M sucrose, 40 mM KCl, 42 mM Ca-EDTA, 20 mM HEPES (pH 6.4),10 mg/ml MagneHis^{TM} Ni-Particles, 5 units/ml β-Galactosidase	1 wt% alginate, 0.45 M sucrose, 40 mM KCl, 42 mM Zn-EDDA, 20 mM HEPES (pH 6.4), 10 mg/ml MagneHis^{TM} Ni-Particles, 5 units/ml β-Galactosidase
Figures S3 and S7	Fluorescence recovery after photobleaching control and calcein permeation control	1 wt% alginate (with 0.1wt% rhodamine tagged alginate) ,0.45 M sucrose, 40 mM KCl 42 mM Ca-EDTA, 20 mM HEPES (pH 6.4) and 10 mgml^{-1} magnetic particles.	1 wt% alginate (with 0.1wt% rhodamine tagged alginate) ,0.45 M sucrose, 40 mM KCl 42 mM Zn-EDDA, 20 mM HEPES (pH 6.4) and 10 mgml^{-1} magnetic particles.
Figure S8	Blank hydrogel control	1 wt% alginate ,0.45 M sucrose, 40 mM KCl 42 mM Ca-EDTA, 20 mM HEPES (pH 6.4).	1 wt% alginate ,0.45 M sucrose, 40 mM KCl 42 mM Zn-EDDA, 20 mM HEPES (pH 6.4).

Table S1: Compositions of the precursor solutions used to produce the variety of hydrogel artificial cells within the main figures.

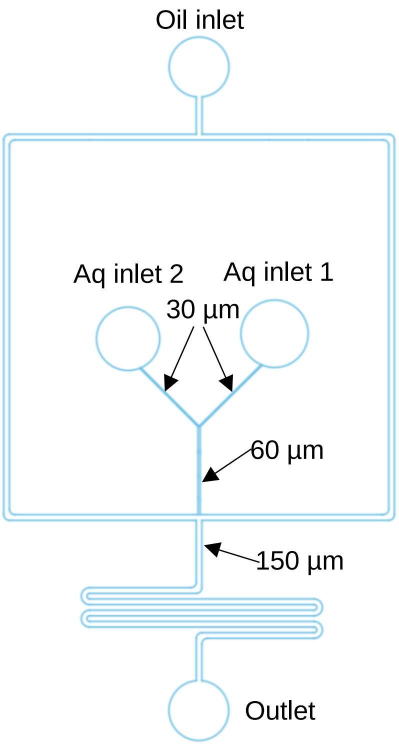

Figure S1: Schematic of the microfluidic chip used. The different channel widths are annotated within the figure. The channel depth was always 100 µm.

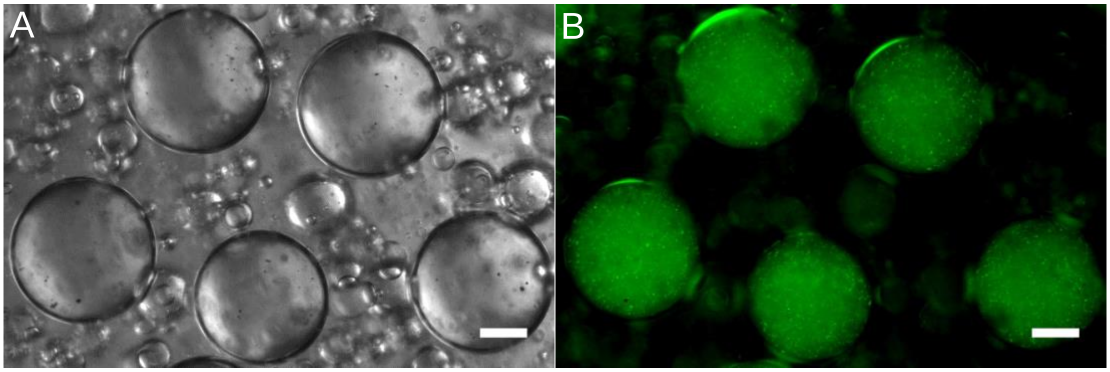

Figure S2: Images of hydrogel artificial cells in the collected microfluidic emulsion. Panel A is a brightfield image while panel B is a fluorescence image. Both images were taken from the collected emulsion present in the Eppendorf tube connected to the microfluidic device before resuspension in aqueous buffer. The fluorescent signal from the vesicles is localised to the aqueous gels. Within the aqueous gels speckling can be seen as in the confocal images indicating gelation has successfully occurred. All images were taken in mineral oil with 5 wt% Span 80. The scale bar on all images is 50 µm.

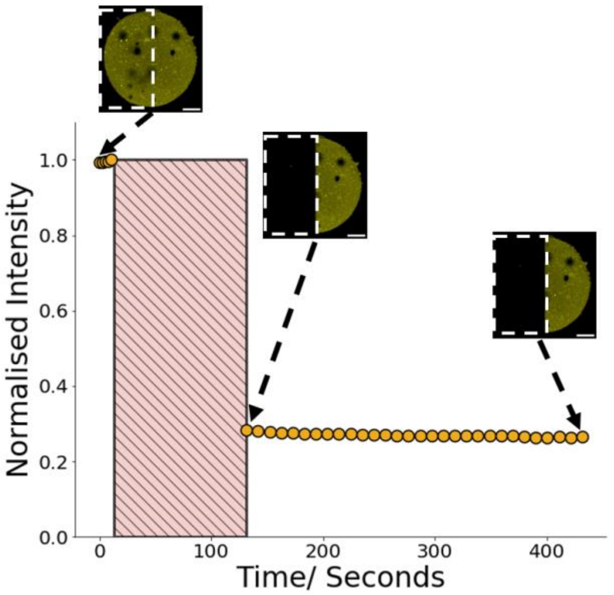

Figure S3: Recovery of a hydrogel artificial cell labelled with rhodamine. A graph demonstrating the fluorescence recovery of a hydrogel artificial cell labelled with rhodamine tagged alginate containing magnetic particles. After bleaching half of the artificial cell for 2 minutes, no fluorescence recovery was observed in the following 5 minutes. The embedded confocal images show the labelled artificial cell before bleaching, immediately after bleaching and 5 minutes after bleaching. The white box indicates the region being bleached. The lack of recovery demonstrates that the alginate is no longer in a fluid state, thus supporting successful gelation of the hydrogel artificial cells. The scale bars are 20 µm.

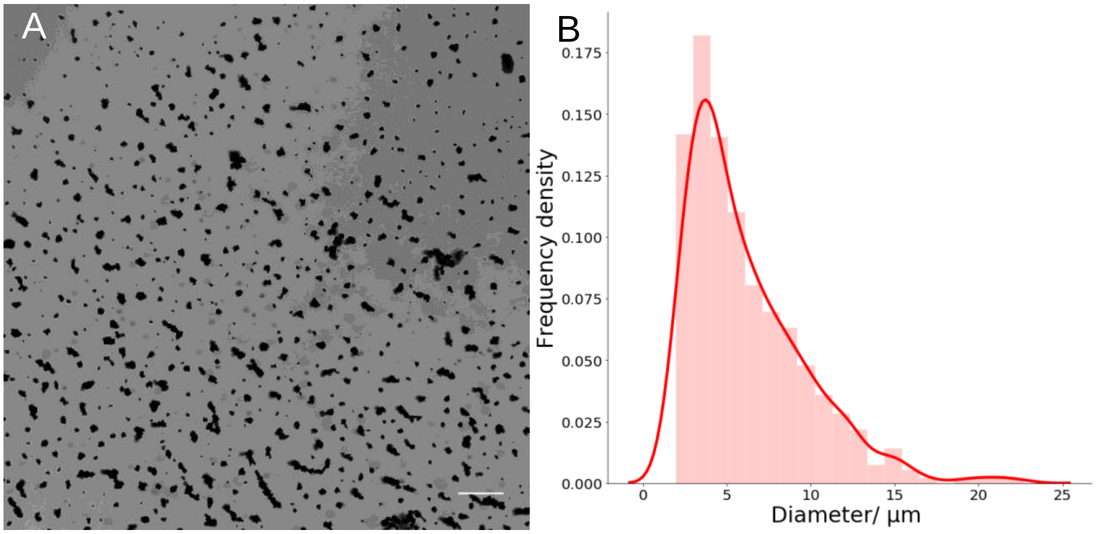

Figure S4: Size profile of the magnetic particles. Panel A is a brightfield image of 0.5 mgml^{-1} magnetic particles in sucrose buffer (0.5 M sucrose, 100 mM HEPES, 100 mM KCl, 20 mM $CaCl_2$ pH 7.4). The scale bar = 50µm. Panel B represents the size distribution of the magnetic particles with an average diameter of 6.2 µm. The larger particles are aggregates of the smaller particles, hence a wide range of sizes (n=882).

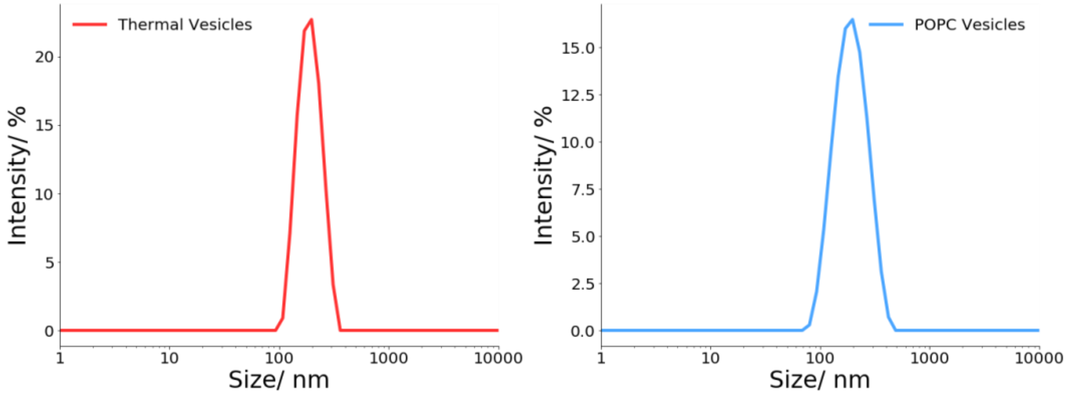

Figure S5: Dynamic light scattering of the vesicle populations before insertion into the hydrogels. The thermal composition vesicles had an average size of 182 nm with a polydispersity index of 0.07 while the POPC vesicles had an average size of 178 nm with a polydispersity index of 0.1. Samples were measured by diluting the vesicles in a 1:10 ratio in sucrose buffer (0.5 M sucrose, 100 mM HEPES, 100 mM KCl, 20 mM $CaCl_2$ pH 7.4).

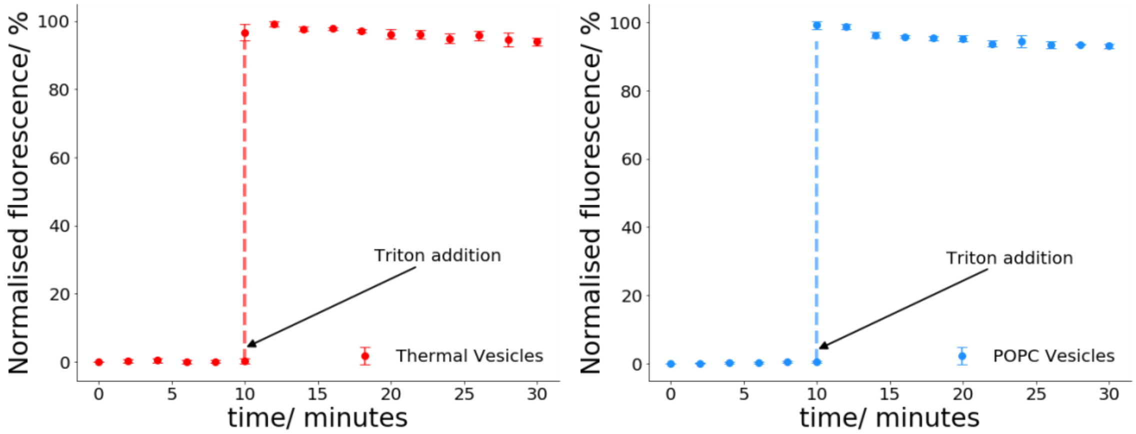

Figure S6: Verifying encapsulation of Calcein dye within different vesicle populations. The vesicles were diluted in a 1:10 ratio in sucrose buffer (0.5 M sucrose, 100 mM HEPES, 100 mM KCl, 20 mM $CaCl_2$ pH 7.4). The samples were monitored for 10 mins before addition of Triton X-100 which caused vesicle lysis and release of quenched calcein. This demonstrates that both vesicle populations were able to successfully encapsulate cargo. The error bars show the standard deviation of n=3 vesicle populations.

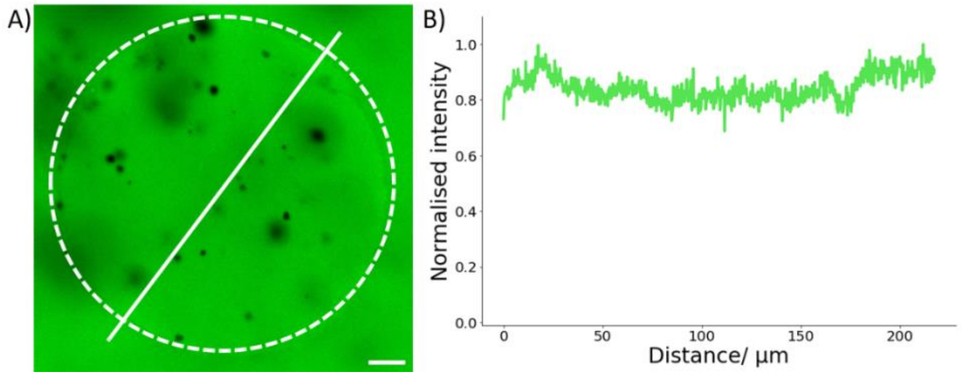

Figure S7: Unencapsulated calcein permeation through hydrogel artificial cells A) A confocal microscope image showing the ability of the calcein to readily permeate through hydrogel artificial cells containing magnetic particle organelles. The border of the hydrogel artificial cell is shown by the dotted line while the straight line indicates the line profile used in panel B. **B)** A line profile through the artificial cell showing that the intensity of the calcein signal is consistent both inside and outside the artificial cell demonstrating that calcein easily permeates into the artificial cells. The image was taken 1 minute after the addition of 0.25 mM calcein. The scale bar is 20 µm.

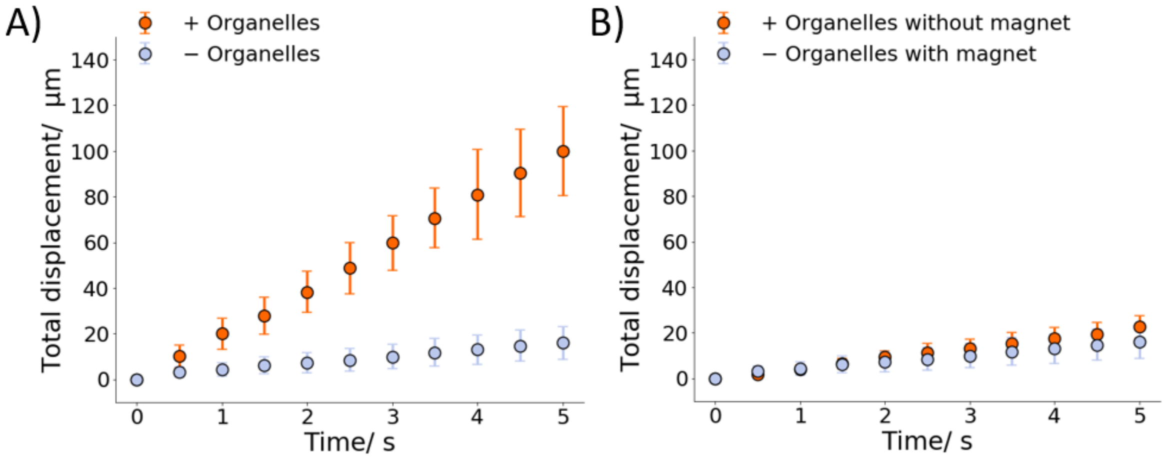

Figure S8: Motility of hydrogel artificial cells without organelles. A) Comparison of hydrogel artificial cells with and without organelles with the magnetic field applied. In the absence of organelles limited motion is observed. **B)** Comparison of hydrogel artificial cells without organelles in a magnetic field to hydrogel artificial cells with organelles in no magnetic field. The total displacement observed after 5 seconds is similar demonstrating that the magnetic field on the hydrogel artificial cells without organelles has no impact. The error bars indicate 1 σ (standard deviation) from an n=7 data set.

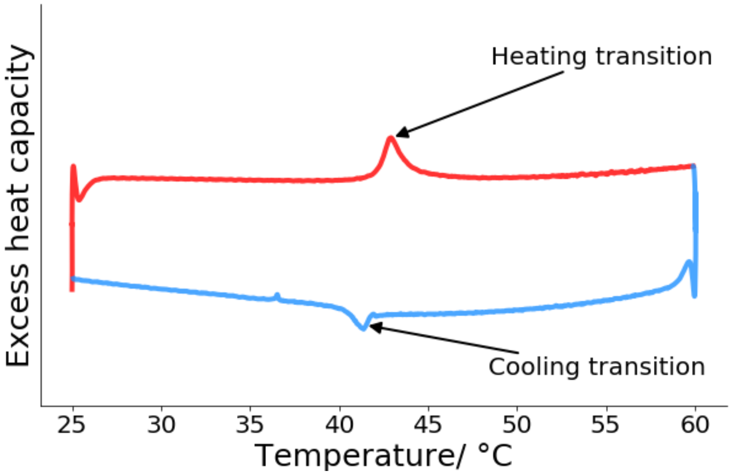

Figure S9: DSC thermograph of the 8:1 DPPC: Cholesterol lipid composition. It can be seen that at 43 °C there is a peak which corresponds to a phase transition from a gel phase to a fluid phase. In an analogous manner on the cooling scan a fluid to gel transition at 41 °C is observed. This demonstrates that the produced vesicles of this composition will also have a phase transition at this temperature and thus are thermoresponsive when heated and subsequently cooled down. The scan rate was 5 °C a minute.

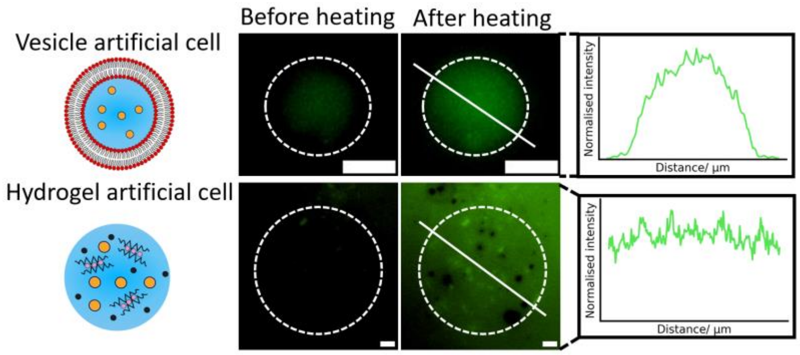

Figure S10: Comparison of hydrogel artificial cells to vesicle artificial cells. Artificial cells comprised of a DOPC lipid chassis with 8:1 DPPC: Cholesterol SUV organelles contained within were compared to the hydrogel artificial cells containing the 8:1 DPPC: Cholesterol SUV organelles. Upon heating to 50 °C an increase in fluorescence is observed in both systems due to cargo release from the thermoresponsive organelles. However, within the vesicle system this increase is confined to the lumen instead of the entire external environment. This demonstrates that the two different systems have different release properties. The dotted circles show the position of the vesicle and hydrogel artificial cells, and the straight lines indicate the line profile use for the respective intensity distance plots. The scale bars are 10 µm for the vesicle artificial cells and 20 µm for the hydrogel artificial cells.

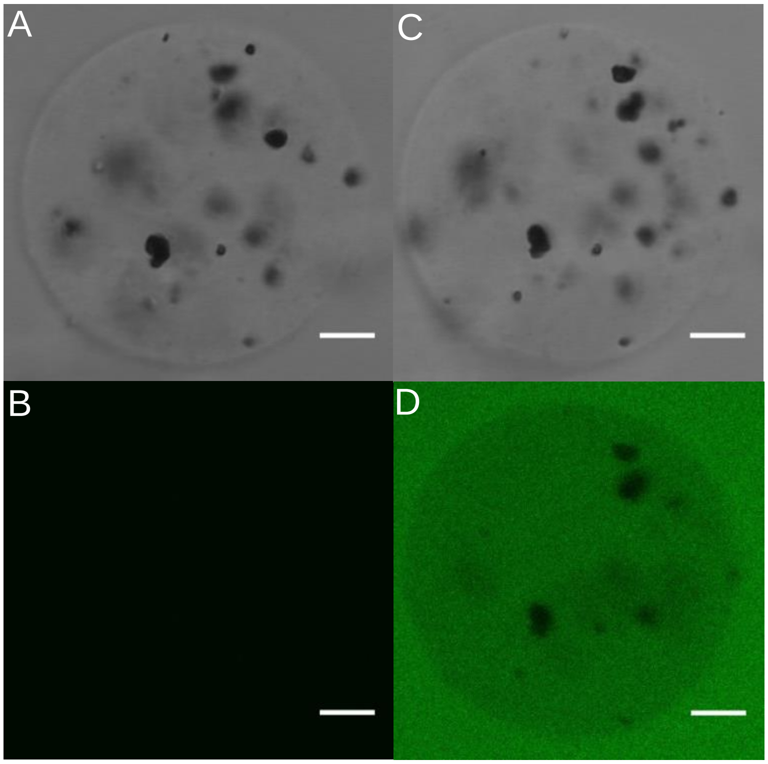

Figure S11: Demonstrating 70 kDa FITC-Dextran diffusion into the hydrogels through confocal microscopy. Panels A and C show brightfield images of a small hydrogel before and 1 min after the addition of a 0.01 mM 70 kDa FITC labelled Dextran. Panels B and D show the fluorescent signal before and 1 min after addition of the Dextran. Upon addition, the Dextran quickly diffuses through the gel demonstrating that the pore size of the hydrogel is larger than the size of the 70 kDa Dextran molecule. As the $sPLA_2$ enzyme and α-Hemolysin monomers are smaller in size than this Dextran (14 kDa and 33 kDa respectively), it would be expected that these substrates freely diffuse into the hydrogel. All images were taken in sucrose buffer (0.5 M sucrose, 100 mM HEPES, 100 mM KCl, 20 mM $CaCl_2$ pH 7.4). The scale bar on all images is 10 µm.

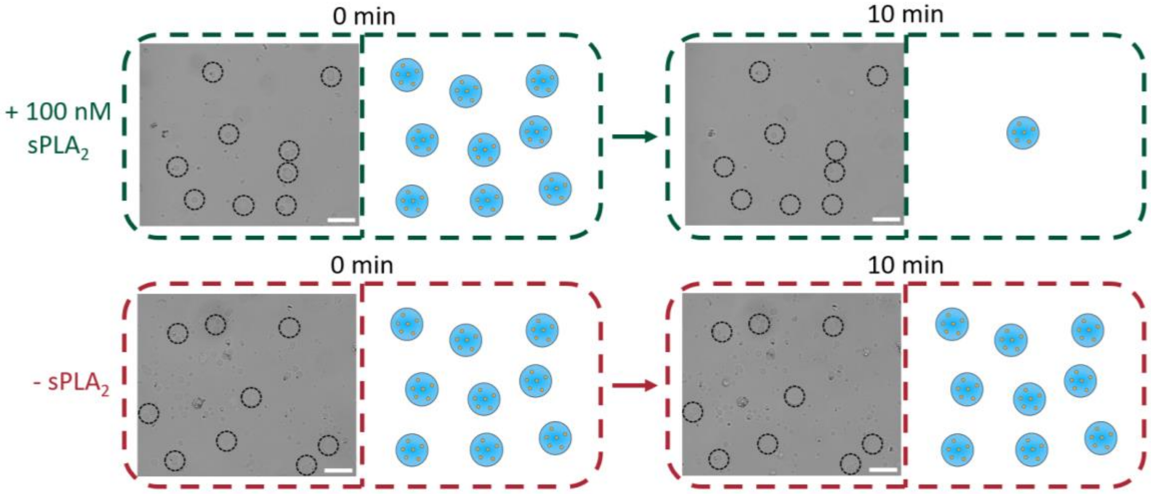

Figure S12: Impact of $sPLA_2$ addition to vesicle artificial cells. Artificial cells comprised of a DOPC lipid chassis with 8:1 DPPC: Cholesterol SUV organelles had 100 nM of $sPLA_2$ added to the solution. Within 10 minutes the $sPLA_2$ lysed the lipid vesicle chassis causing the embedded organelles to be released into the external environment. The hydrogel artificial cells are not lysed with $sPLA_2$ hence demonstrating their utility to be a stable chassis in conditions that would cause the failure of other predominantly lipid based artificial cell systems. The dotted circles are used to highlight some of the positions of the vesicle artificial cells and the accompanying schematics represent the change in population size with/ without $sPLA_2$ addition. The scale bars are 50 µm.

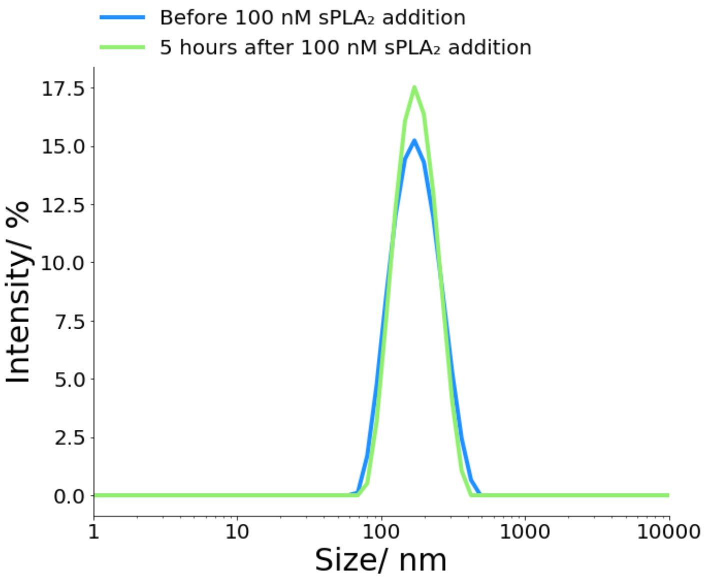

Figure S13: The impact of $sPLA_2$ on SUV stability. The addition of $sPLA_2$ on POPC vesicles led to no observed micellization after 5 hours. This demonstrates that calcein leakage from the addition of $sPLA_2$ is occurring through defects in the bilayer and not also through the conversion of the vesicles to micelles. Samples were measured by diluting the vesicles in a 1:10 ratio in sucrose buffer (0.5 M sucrose, 100 mM HEPES, 100 mM KCl, 20 mM $CaCl_2$ pH 7.4) and adding 100 nM of $sPLA_2$.

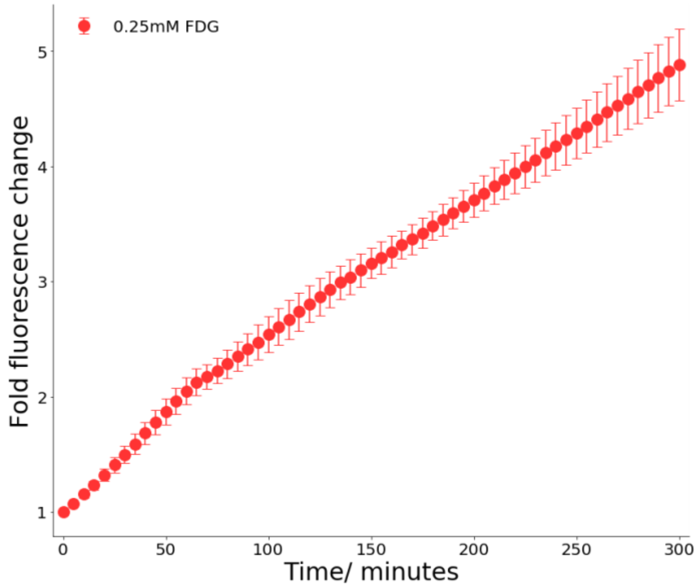

Figure S14: Encapsulation stability of β-Galactosidase within the hydrogels. After 24 hours at room temperature the suspended hydrogels containing β-Galactosidase were centrifuged again to produce a pellet and resuspended in fresh buffer (0.5 M sucrose, 100 mM HEPES, 100 mM KCl, 20 mM $CaCl_2$ pH 7.4). The hydrogels were then incubated with 0.25 mM of FDG for 5 hours and an increase in fluorescence signal was seen indicating that β-Galactosidase was still present within the hydrogel system. This illustrates that complete leakage of the β-Galactosidase from the hydrogels into the buffer had not occurred within a 24-hour period and confirms that the enzymatic reaction between FDG and β-Galactosidase is occurring within the produced hydrogels. The error bars show the standard deviation of n=3 hydrogel populations.

SI video descriptions

Video S1- A timelapse demonstrating hydrogel production using a microfluidic device.

Video S2- Brightfield Z stack showing magnetic particle localisation within a hydrogel.

Video S3- Fluorescent Z stack showing POPC vesicle localisation within a hydrogel.

Video S4- Timelapse of hydrogel movement before and after magnet application.

Video S5- Timelapse of calcein release from a hydrogel using 100 ngμL^{-1} of α-Hemolysin.

Video S6- Timelapse of conversion of FDG to Fluorescein using β-Galactosidase embedded in a hydrogel. The FDG is released from surrounding vesicles with 100 ngμL^{-1} α-Hemolysin.
